# “I Think the Teachers Should Really Connect More With the Students”: The Influence of Systemic Racism, Inequity, School, and Community Violence on Connection for High School Students Who Are Suspended or Expelled

**DOI:** 10.1177/0044118X231226396

**Published:** 2024-01-25

**Authors:** Jane E. Sanders

**Affiliations:** 1 King’s University College at Western University Canada, London, ON, Canada

**Keywords:** suspended, expelled, discipline, trauma, adversity, ACEs, school violence, racism

## Abstract

The objective of this constructivist grounded theory study was to understand the experiences of students who have been disciplinarily excluded from school. Fifteen students (male, *n* = 11; Black, *n* = 10; having special education needs, *n* = 9) and 16 multidisciplinary staff in Ontario participated. Students experienced high rates of expanded adversities, including school and community violence, systemic racism and inequity. The importance of connection wove throughout the data; however, three themes were found to block connection: unacknowledged impact of adversity, a climate of fear, and the disproportionate impact of limited resources. Trauma-informed culturally attuned approaches that focus on the disproportionate impact of adversity and school discipline at the point of a disciplinary response, and throughout a student’s educational experience, are essential.

Adverse experiences and resulting trauma can significantly affect a child’s social, emotional, and neurological development (Yehuda et al., 2015). This in turn can negatively impact academic outcomes including performance, engagement, attendance and graduation (Fry et al., 2018). Moreover, inequity is produced and maintained through structural and interpersonal oppression often involving violence, abuse, exploitation, exclusion, and humiliation (Wilkin & Hillock, 2014). Therefore, the impact of adversity is disproportionate based on a range of factors including race, Indigenous status, gender, poverty, sexuality, and (dis)ability (Holmes et al., 2016). Likewise, school suspension and expulsion (exclusionary discipline) disproportionately impacts students who are Black, Indigenous, mixed race or Middle Eastern, male, have special education needs, or are from lower income households (James & Turner, 2017; Leung-Gagné et al., 2022). These groups are more likely to be suspended, suspended for longer, or expelled than White students (Balfanz et al., 2015). However, the relationship between early adversity and discipline receives very little attention (Sanders et al., 2023). This lack of attention colludes with an ongoing failure within education to adequately understand, address, and support certain students who are coping with experiences of adversity, and too often disproportionate discipline (Morris, 2016; Singletary, 2020).

Trauma-informed care elucidates how behavior, relationships, spiritual, and mental health are impacted by violence and adversity regardless of whether we are aware of these experiences (Fallot & Harris, 2001). Trauma-informed approaches re-interpret coping such as substance misuse, extreme anger, or self-harm as an appropriate response to adversity, foster an acknowledgment of the role of adversity and a shift in understanding and support within systems, including education (Wilkin & Hillock, 2014). Healthy relationships and connection, which include understanding the lives of students and the meaning of their behaviors, are not only critical for school success but are key within schools with low suspension rates (Anyon et al., 2018; Ekstrand, 2015). The objective of the current constructivist grounded theory study was to understand experiences of students who have been disciplinarily excluded from school.

## Understanding Trauma and Adversity

Trauma refers to the symptomology or impact of a distressing or disturbing experience, such as a car accident or a natural disaster, witnessing harm or being harmed by someone (Santiago et al., 2013). Adversity refers to experiences that are harmful or threatening and can lead to trauma, or the absence of stimulation required for typical human development (Koss & Gunnar, 2018). While the terms trauma and adversity are understood distinctly, adversity can lead to trauma and experiences of adversity may or may not have an impact regardless of whether the response is recognized or categorized as trauma.

Specific forms of adverse childhood experiences (ACEs) were identified in the 1990s as negatively impacting long-term health (Felitti et al., 1998). These conventional ACEs were defined as: psychological, physical, or sexual abuse; physical or emotional neglect or abandonment; death of a parent; violence against mother; parental separation or divorce; living with caregivers who misuse substances, experience mental illness, suicidal behavior, or were ever imprisoned. These conventional ACEs focused on experiences in the home and included predominantly White, middle-class samples. ACEs have since been expanded to include experiences outside the home and those that disproportionately impact diverse populations: peer victimization, isolation and peer rejection, close network member being seriously ill or attempting suicide, exposure to community violence, low socio-economic status, racism, living in an unsafe neighborhood, and having lived in foster-care (Cronholm et al., 2015; Turner et al., 2020).

## Disproportionate Discipline and Adversity

After the removal of zero tolerance from Ontario legislation in 2008 and the institution of progressive discipline, restorative practices, and school based mental health, the rate of suspension decreased from 5.49% of students in 2007 to 2.67% in 2017 and expulsion decreased from 0.09%, to 0.02% (Government of Ontario, 2017). This is in contrast with the 5% suspension rate in 2017/18 in the U.S. where the bulk of the research is conducted (Leung-Gagné et al., 2022). However, disproportionate discipline remains unrelenting as Black students in Ontario, were expelled at 4 times their representation and Indigenous students at over 3 times (James & Turner, 2017).

Owens and McLanahan (2020) found that unequal support of Black compared to White students in the U.S. was a significant factor in the disproportionate rate of exclusionary school discipline. Students perceived as well behaved did not experience this unequal support. Concerningly, Mayor (2022) found that young students of color in Ontario were over-surveilled, quickly labeled as “bad” or “dangerous,” and were less likely than White students to be viewed as experiencing adversity. Polyvictimization increases the potential for negative outcomes including academic difficulties and increased risk of exposure to subsequent adverse experiences, which can then disproportionately escalate in adolescence (Andrews et al., 2019). Therefore, the reinforcing aspects of early racial inequity have cumulative implications for students (Owens & McLanahan, 2020). Inequity experienced through disproportionate exposure to adversity is further exacerbated when the impact of adversity is unacknowledged, and students are treated as perpetrators of violence and supported in ways that are not equal to others.

## Connection in Schools

Student-teacher relationships are known to influence school engagement and achievement and this influence continues throughout life (Valdebenito et al., 2019). Attending an elementary school with at least one guidance counselor reduces the odds of being suspended by 22% (Mittleman, 2018). It is concerning, therefore, that students’ reports of receiving the support they need declined from 85% in grade 4 to 66% in grades 9 to 12 (TDSB, 2018). Additionally, while only 36% of educators expressed confidence dealing with physical violence there has been an increase in violence against educators from 7% in 2005 to 54% having experienced violence at some point in their careers in 2018 (Santor et al., 2019). Moreover, the top 5% of educators who refer students for office discipline are likely to be early career teachers and would benefit greatly from additional support, as would the students they teach (Liu et al., 2023).

## Theoretical Foundation

Grounded theory is rooted in symbolic interactionism which views our understanding of another’s actions and our response in context to the meanings we ascribe to these actions, co-constructing our view of self, situations and the broader society (Charmaz, 2014). Intersectionality fostered a critical examination of the ways that oppression can be compounded based on students’ ascribed social identities including race and gender within a historical and current context of systemic inequity, hierarchy and power (Crenshaw, 1991). An intersectional perspective grounded in expanded adversity research therefore confirms the role of systemic inequality in creating disproportionate exposure to adversity. Ecological theory further contextualized individual development within the multiple layers of the environment (Bronfenbrenner, 1979).

## The Current Study

Constructivist grounded theory was employed to develop preliminary theory about complex interactive social processes related to adversity and disciplinary exclusion. The objective of the current study was to understand the experiences of students who have been disciplinarily excluded from school and center their voices, along with those who support them, in this discussion. The following research questions guided this analysis: 1) How do experiences of expanded adversity impact students? 2) How can schools support students with similar experiences?

The current study focuses on several gaps in the literature. Few studies have entailed speaking with students who have been disciplinarily excluded, and fewer still explicitly consider adversity or trauma (see Bell 2019; Crosby et al. 2018; Haight et al. 2014). Additionally, the relational aspects of disciplinary exclusion are poorly understood (McGrew, 2016). Universal policy changes that decrease disciplinary exclusion generally, are ineffective in changing the level of disproportion in school discipline (Valdebenito et al., 2019; Welsh & Little, 2018). Finally, research on the pathway from adversity exposure to disciplinary exclusion is needed to inform interventions (Loomis et al., 2020).

## Methods

Through grounded theory methodology novel generation of theory is derived directly from the data. The focus is on explaining patterns and informing future research and practice, particularly appropriate when little is known within a substantive area (Charmaz, 2014; Glaser, 2014). In constructivist grounded theory, there is an emphasis on the interpretive and constructivist aspects of research, which means the researcher actively engages with the data and helps to construct theories that are grounded in the data (Charmaz, 2014).

Participants were recruited through Caring and Safe Schools (CSS) classrooms in two participating school boards serving racially, culturally, and socioeconomically diverse, urban (city of over one million) and urban emergent areas of Southern Ontario. Students on long-term suspension or expulsion in Ontario are provided an academic and behavioral support program through their CSS department. In rare circumstances, a family and school board will agree to a student attending CSS without disciplinary exclusion. CSS programs offer a secure and separate location, small classes, a high teacher-to-student ratio, child and youth workers, social workers, psychologists, and guidance counselors. Students receive one-to-one support at their individual pace. Students and staff participate in daily group check-ins and check-outs, prepare food, eat together, and play games over lunch. Staff have regular contact with families and call missing students daily to check in and encourage attendance. Students stay in CSS programs until completion of the suspension, or if expelled, considered ready to attend a typical or alternate setting, averaging one semester to 1 year.

### Participants

All students, staff, and caregivers involved in CSS programs were eligible to participate in the current study (*n* = 31). Despite active recruitment efforts only one caregiver was interviewed, too small a subsample for inclusion. Student participants (*n* = 15) were aged 14 to 19, most identified as male (*n* = 11), Black (*n* = 10), or having an individual education plan (IEP) (*n* = 9) (see Table 1). The CSS staff sample (*n* = 16) represented the range of CSS professionals (see Table 2). The CSS staff were included as a form of triangulation providing data from more than one type of observer. Given the small representation of certain subgroups (e.g. female and Indigenous students), demographic information is not attached to participant quotes to protect confidentiality. Ethics approval was obtained through the supporting university and both participating school boards. Consistent with constructivist grounded theory the focus on violence and adversity originated with the principal investigator’s (PI) over 25 years of experience in child, youth and family therapy, trauma assessment and treatment, and research in school boards.

**Table 1.** Student Participants (*n* = 15).

Characteristics	Sample
Gender	
Female	4
Male	11
Race	
Black (including 2nd and 3rd generation descendant from African or Caribbean country)	10
South Asia/East Asia/Middle East	3
Self-identified White and 3rd generation Indigenous	1
White	1
Age	
Mean age (standard deviation)	16.6 (*SD* = 1.5)
Grade	
Grade 9	1
Grade 10	3
Grade 11	3
Grade 12	7
Individual education plan (IEP)	
IEP	9
No IEP or did not know	6

**Table 2.** Staff Participants (*n* = 16).

Characteristics	Sample
Profession	
Child and youth workers	4
Guidance counselor	1
Psychologist	1
Social workers	3
Teachers	6
Vice-principal	1
Race	
Black	5
South Asia/East Asia/Middle East	2
White	9
Gender	
Female	10
Male	6

#### Recruitment

The PI introduced the study to 30 students and 25 staff of CSS programs. Informed consent packages were distributed to guardians through students and additional packages were left for absent students. Student and parent participants received $25 gift cards and CSS staff received $5 gift cards.

### Data Collection

Data were gathered through semi-structured, in-depth, face-to-face interviews, from October to April 2018/19, lasting 60 to 90 minutes, and memo writing before, during and following data gathering, analysis, and writing. Interviews were transcribed by the PI, by a research assistant, or by a professional transcription service. NVivo 12 (QSR International Pty Ltd., 2018) was used to organize the data and analysis.

Researcher biases and prior knowledge were reflexively examined and included in the analysis through constant comparison (Breckenridge et al., 2012; Glaser, 1998). Memos were used to explore the researcher’s perceptions, experiences, emotional reactions, and existing knowledge, and to document engagement with other researchers. The PI consulted throughout all phases of the research with a team of diverse researchers in terms of gender, race, ethnicity, age, and research specialization related to adversity, marginalized communities, and education. This clinical and research experience provided a form of prolonged engagement and accountability. Prolonged engagement was furthered through 7 months of data collection from October to April, involving 31 engagement, recruitment or interview visits at each of the six locations.

Interviews began with open-ended questions to elicit both staff and student perspectives on students’ experiences of school, community and family, and the influence of these experiences on students’ pathways. Toward the conclusion of the student interviews the PI verbally reviewed a demographic questionnaire of student’s age, gender, race/ethnicity, credit count, grade, ever assigned an IEP, suggested or identified as having a mental health concern, offered or received school or community supports, extracurricular activities, and strengths and future goals. All participants were asked to reflect on the overrepresentation of specific populations within disciplinary exclusion, eliciting knowledge of systemic inequity. Adversity was defined and participants were asked whether, and approximately how many forms of, adversity students in the program experienced. Students were asked about their own experiences and staff were asked to reflect generally across CSS students. This combination of semi-structured interviews and structured adversity questions captured both complex narratives and the number of ACEs that participants may not have considered, be reluctant, or unable to detail. Students were asked what information or advice they had for schools to support students who had similar experiences to theirs.

### Data Analysis

Student interviews were prioritized for initial line-by-line coding. Codes were compared, synthesized and analyzed. Experiences of adversity consistently emerged across the first few interviewers and guided ongoing data collection and analysis. Initial codes were reviewed, and constant comparative analysis was applied within and across interviews (Glaser, 1998). Through focused coding the in-vivo code “connection” inductively emerged as the most effective way to support youth. The unacknowledged impact of adversity, fear, and limited resources were identified as barriers to connection. The constant comparative method guided the coding and analysis of the first 14 staff interviews. Theoretical saturation was reached at 10 student and 14 staff interviews. Five additional student interviews and two staff interviews ensured saturation of codes and provided a form of member checking, to further check and refine categories, to test the theory in the field, and ensure fit and understandability (Charmaz, 2014). Four staff and four student interviews were independently peer coded by two PhD candidates (O’Connor & Joffe, 2020) and incorporated through constant comparison to foster reflexivity and dialogue, trustworthiness and fit of the emerging theory. Trustworthiness was further augmented by thick description of the findings (Charmaz, 2014).

## Results

The importance of connection wove throughout the data. Every student wanted teachers and school staff to connect with them, get to know them, and demonstrate they care about their success. When asked to share advice for teachers on supporting students who are struggling students were clear, “I think the teachers should really connect more with the students. I don’t know. Like, ask someone how they’ve been and shit. Like, how is it outside. About their days and stuff” (ST15). Participants felt this connection at school had been lacking before being disciplined.

### Factors That Blocked Connection

Three interconnected factors emerged as blocking connection: unacknowledged impact of adversity, fear, and limited resources.

#### When Adversity Is Unacknowledged Connection Is Limited

All participants concurred that CSS students experienced high rates of multiple and diverse forms of adversity. Fourteen of the 15 student participants identified experiencing at least two forms of adversity, and 7 identified five to seven distinct forms, a particularly concerning rate of polyvictimization. Students shared experiences of sexual abuse, death of a parent or friend, caregiver experiencing mental health concerns, being exposed to conflict in another country, family conflict, and financial difficulties. These often intertwined in complex ways, for example, “my mom . . . wasn’t working for a couple of years . . . she has depression and very bad anxiety” (SD08). Another participant was 10 years old when he was assaulted by a 14-year-old “he knocked out one of my teeth . . . My nose was bleeding, and I had a black eye . . . Yeah, he punched me in the face a whole bunch” (ST10). Most relayed witnessing or experiencing violence, including weapons, both inside and outside of school. Such expanded forms of adversity including systemic racism, inequity, and school and community violence emerged as particularly impactful common experiences (Sanders et al., 2022).

CSS staff also reflected on the importance of adversity on students’ school success as “impacting how they’re doing in class, in school, in education, in their learning” (SF06). Despite the extent and impact of exposure to adversity, staff believed it was rarely acknowledged, “we [don’t] really recognize or acknowledge some of the things the students that we see in Safe Schools . . . go through” (SF10).

Staff gave examples that illustrate the importance of acknowledging adversity, “she does not care about the credits if she’s not living anywhere. The fact that she actually made it to school . . .” (SF07), and

during the day, he’s fine. Towards the end of the day he becomes really moody. Really quiet and he said, “my priority is safety. How can you guys ask me to focus on this and I don’t know what’s going to happen when I am out there.” (SF10)

CSS staff recognized the importance of acknowledging expanded ACEs to understand student experiences and the ways they are impacted, “I think interrelations, understanding our students, understanding in a holistic sense the value of recognizing the challenges students face through adversities . . . And not thinking that students leave that at the door when they come into class” (SF06).

Students felt there was particularly insufficient acknowledgement of adversity occurring in schools. They felt that too often teachers turned a “blind eye” to the violence and they wanted to feel safe at school (SD07). They felt that schools rarely addressed bullying and school violence as adversity and did not support those involved as vulnerable. One student articulated, “I’m not blaming the school for my actions or anybody else’s action whenever they fight. But . . . they could do a lot more” (SD12). Many described being targeted in grade nine. For example, one described being “jumped” by a group of peers in grade nine, “even though I didn’t do anything. I was suspended” (SD14). The student participants felt they were rarely listened to and were insufficiently supported to deal with difficulties, particularly bullying and violence, “I feel like at least if the teachers didn’t always target me and try to like suspend me when I was younger . . . Like I actually, I went to go and look for help and stuff” (ST07). CSS staff supported this perspective, “no one is taking the time to make the connection . . . it’s just being neglected. It’s almost like they’re not there . . . teachers aren’t catching the connection that maybe there’s something going on” (ST16).

#### In a Climate of Fear Connection Is Limited

Teachers were seen as scared to ask students how they are doing, blocking connection, and understanding, potentially limiting acknowledgement of adversity, and effectively leaving students on their own to cope with safety concerns. One staff labeled the current environment a “climate of fear” for both students and staff. According to this staff, “we live in societies that we know that violence happens in schools. We’ve seen it so many times . . . it’s traumatizing for all involved . . . Everybody is traumatized. So, yeah, that’s what I mean about a climate of fear . . .” (SF15). This fear and systemic bias directly affected students’ relationships and connection with staff. As one student explained, “people were saying I had a weapon on me . . . then all the teachers heard about it, so I know the teachers started keeping a distance away from me. They got scared, right?” (SD09). Another student described the change at school after an assault prompted police to be placed in their school,

And now [teachers] would just talk to you like you are a waste youth. And you don’t want to hype up now because if you hype up teachers would feel some kind of way, threatened or some kind of way, endangered. (SD07)

Many students felt that disciplinary responses were based on the level of threat they were perceived to pose rather than the actual incident, “It’s the way they look at people. If they look at you and they think that you are a danger . . . People are very biased” (SD10). This student had experienced an extremely high level of polyvictimization and spoke about the way that impacted their focus in school and their relationships with teachers. Another student spoke about teachers being scared and how that affected their relationships in schools, “yeah. just assume, assume that like I’m a troublemaker because of what, how I look, how I present myself” adding, “sometimes, like you can tell, you can tell when somebody is scared of you, you know?” (SD03). Participants identified how systemic racism across society manifested in schools, as one student stated,

I think it’s just like . . . how people are seeing you . . . I’m not trying to say anything bad, but it’s like, all bad communities, they feel like all the bad stuff comes on young boys, [students of] colour, and stuff. I feel like even in school, in person, it’s like teachers were scared to ask them how they’re feeling because it’s how they dress, how they speak, how they do stuff. (SD09)

As CSS staff observed, “the news media plays into that in terms of highlighting the negative . . . the emphasis is . . . on the area that they occur” (SF14). Students who were perceived to be exposed to “bad communities” were viewed with fear which limited teacher-student interactions and ultimately connection. Therefore, not only does adversity affect an individual’s social, emotional, and neurological development, social identities are imposed on certain students based on their social location, which influences how they are viewed, approached, and supported within systems such as education.

#### When Resources Are Limited Connection Is Limited

Allocation of resources ranges from brief exchanges in the hall encouraging a student to return to class, to class size, and staff training (Owens & McLanahan, 2020). Student and staff participants recognized that staff in larger settings have limited resources to distribute among students with varying educational needs. One student asserted, “too many kids. The teacher more focused on the ones that really, really needed help . . . if you were getting your work done and just were kind of struggling, he would . . . brush it off” (SD08). Available resources directly influenced the pathway to CSS, as one student articulated, “That’s why I’m here . . . I couldn’t concentrate, because there was like 30 students talking and just asking questions” (SD13).

In contrast, student participants recognized that the resources available at CSS fostered connection within CSS and they were proud they were earning credits and feeling success. One student observed that “every teacher here they all have the skills of a social worker kind of . . . They always ask every day, ‘how was your weekend? Good. How are you feeling? Do you want to talk?’. . . everyone, literally everyone” (SD04). CSS staff described their available resources such as multidisciplinary knowledge, skill, and a high staff-to-student ratio, which enabled connection with students. CSS staff noted that the way they approach students who appeared to be managing adversity would be difficult in a mainstream setting given the typical resources, “you’re having trouble working . . . Oh, let’s go talk . . . They can’t do that in regular schools, but we can here” (SF11). The impact of this was profound as in an environment of limited resources eventually students stopped looking for support: “so if no one’s reaching out to them, they’ll just get missed. And they’ll feel like no one cares . . . a thousand kids in the school. You got 30 kids in a class. It’s hard to . . . gauge where every kid is at” (SF12). Students identified the importance of increased support, and even of being held accountable, and this communicated that school staff cared, for example: “The teachers weren’t necessarily bad, but nobody was on you. You could skip class, leave, they won’t call your parents” (SD14) and “nobody cares if you do [an assignment]. If you fail, you fail you know” (SD03). Students wanted staff to try harder to get to know them, to connect with them, and to care if they were attending and doing work.

### An Interactive Social Process

The unacknowledged impact of adversity, a climate of fear, and limited resources appeared to influence the connection between students and teachers, and ultimately student success:

A kid comes in, looking a certain way, acting a certain way, teachers are afraid and immediately set down with whatever version they have and the kid’s trying to give his side. They’re not listened to, no one hears them. Now, you’re suspended and you come here. (SF16)

This interactive social process interfered with the identification of students who needed support. The significant impact of exposure to violence is missed when adversity is not acknowledged as traumatizing for certain youth, “it’s how racism plays out sometimes and the lack of understanding around people who are disadvantaged . . . generational trauma, children who come from poverty, how things manifest in education” (SF15). CSS staff expanded on the need to acknowledge what students are coping with, particularly students of color, “You have to see that there is a difference, then you can understand that the student walks through society already being targeted. If you know that, you’re going to change your behaviour, because you understand it” (SF16).

When connection was blocked, students stopped talking to people about the adversity they had experienced, “I keep it to myself” (SD09). Students were clear that their reluctance to talk about experiences of adversity was reinforced by the lack of response discussed earlier, as one student described,

I’ve had to deal with things outside of school. At one point, I think, I used to talk to people about it, but then because nothing ever changed, so, then, slowly I would stop talking to people totally. So, I just keep it all inside and just don’t talk. (SD11)

For others, asking for help could increase experiences of bias. One staff spoke about a student who was worried about a friend who

was shot two days ago . . . not only are they sort of holding this and then be expected to be students in a class, they don’t feel they can even talk about it because then they’ll become the person that is to be afraid of rather than the person who’s afraid. (SF10)

In the absence of effective support to deal with adversity, students spoke about a way of presenting that denied any vulnerability as a strategy for coping (Sanders, 2022). Importantly, this strategy for coping with adversity emerged from a perceived lack of response from schools and society to keep children safe.

The students in this study were clear, they wanted to feel that teachers cared about their success and therefore about them, to make efforts to “connect with their students more” (SD14), and get to know them, as “after you get to know me . . . they wouldn’t be like scared like that” (SD03). This would foster connection and ultimately student success as this same student said, “Like once you get to know me, like I do the work more, you know, because . . . the teacher start to be friends, you know” (SD03).

## Discussion

The emergent grounded theory indicates that the level of exposure to adversity among disciplinarily excluded students is rarely acknowledged, both in research and in practice (Sanders et al., 2022, 2023). This lack of acknowledgement results in a failure to understand and address the needs of students with trauma histories, too often resulting in the application of disciplinary actions and exclusion from school (Delale-O’Connor et al., 2017). The findings of the current analysis suggest that this lack of acknowledgement, combined with a climate of fear and limited resources, blocks connection. Healthy relationships and connection in school includes understanding the lives of students (Anyon et al., 2018; Ekstrand, 2015). Moreover, this interactive social process interferes with identification of students who need support. The findings suggest that certain students are not viewed as traumatized by their experiences, aligning with biased perspectives of these students as perpetrators of adversity rather than experiencing adversity (Sanders, 2022; Sanders et al., 2022; Thompson & Farrell, 2019). When the emergent theoretical process occurs in a social context of inequity and disproportionate exposure to adversity, connection is disproportionately blocked for certain groups of students. For example, school professionals are less likely to recognize adverse experiences for students of color (Mayor, 2022). Staff and students in the current study noted how social stereotypes, systemic racism, and inequity across society influenced acknowledgement of adversity. Moreover, given the general emphasis on conventional ACEs, there is a particular risk that those who are coping with expanded forms of adversity, notably systemic racism, inequity, and school and community violence will not receive trauma-informed support (Sanders et al., 2023).

When adversity is unacknowledged, access to appropriate resources is limited, reinforcing for students that they must cope independently, further blocking connection. Students in the current study were clear that they felt there were few resources available to them through statements such as “too many kids . . .” Disproportionate access to resources, including teacher attention, has a cumulative effect and the way that teachers make sense of student presentations determines discipline choices (Liu et al., 2023; Sanders et al., 2022). Because people act in response to how they view a situation (Charmaz, 2014), teachers and administrators must attend to their perceptions of students and how these perceptions guide their interactions, which students they support and who they shrug off due to resource pressures. Implicit bias includes perceptions (conscious or unconscious) of youth based on race and culture (including SES or gender) as well as expectations of student presentation as interpreted by middle-class, often White, teachers (Welsh & Little, 2018). When viewed from a privileged worldview, coping strategies are easily missed and misunderstood (Sanders, 2022).

Consistent with the current findings, extant research found that disciplinarily excluded students identified that teachers saw “badness” as a pervasive character trait, rather than momentarily related to an incident, which in turn, negatively influenced the ways teachers consequence students (Kennedy-Lewis & Murphy, 2016). Office referrals are generally the first step in the disciplinary process and are driven by subjective reasons like interpersonal offences and perceived defiance (Liu et al., 2023). The racial gap in office disciplinary referrals is doubled by a small group of the top 5% of teachers, often early career, who make these disciplinary referrals (Liu et al., 2023). Individuals create meaning and respond based on how they interpret the actions of others (Charmaz, 2014). Where there is ambiguity about the required discipline in a certain situation, perceptions can sway an administrator or teacher in determining if someone is dangerous and threatening or harmless and having a bad day (Simson, 2014). In turn, being identified as “bad” can elicit responses, reactions, and resistance that reinforce negative perceptions in some situations (Kennedy-Lewis & Murphy, 2016). In contrast, school engagement and connection are associated with a higher sense of well-being, lower rates of “delinquent” behaviors among students, and greater academic success (Romano et al., 2015). High-quality relationships with school, family and community are associated with lower levels of “problematic behaviours” among students who have experienced maltreatment (Wilkinson et al., 2019).

All participants in the current study were concerned about violence exposure in school. It is important to contextualize these findings within the seven-fold increase of violence against educators over 12 years in Ontario noted above (Santor et al., 2019). Moreover, this violence is gendered in that over 85% of educators who experience harassment and violence identify as women (Santor et al., 2019). With increasing violence in school settings, both students and teachers are affected.

### Implications

The emergent theory is built on the finding that students who have been suspended or expelled experience multiple and diverse forms of adversity. Firstly, students and educators must be safe at school and in the community. Secondly, the current study highlights the importance of connection to the success of all students. Removing the identified barriers to connection can include a fulsome trauma-informed and culturally aware acknowledgement of the life experiences of students. Trauma-informed and culturally attuned approaches that focus on adversity including inequity, antiracism, and integration of the impact of antiblackness and anti-indigeneity at the point of a disciplinary response and throughout a student’s educational experience are important (Elmesky & Marcucci, 2023). Education must be appropriately resourced to support educators, particularly early career educators, with trauma-informed training and ongoing support to critically understand how students and staff are shaped by social, cultural and historical contexts (Marcucci & Elmesky, 2023). Ontario educators have called for programs on social-emotional learning, non-physical intervention, better access to mental health supports, educational assistants, earlier identification of student needs, educational resources, clear and consistent policy, and training for administrators (Santor et al., 2019). Educators who have knowledge and space to critically self-reflect and are aware of the profound impacts of social bias are more likely to correct themselves when influenced by biased perspectives of students fed by socially constructed views of who students are and who they want to become (Pope et al., 2018). Finally, ongoing research is needed on trauma-informed, culturally aware practices that focus on connection and address resource limitations to support students who are at risk of being suspended or expelled. Further study is also needed on the prevalence of adversity and the role adversities may play in disproportionate disciplinary exclusion.

### Strengths and Limitations

While the current qualitative study does not strive toward generalizability, the findings are consistent with extant literature and provide direction for policy and future research. This study lacks parent/guardian perspectives and while this may have strengthened the study, the current analysis is founded on the rarely heard student voices who know their story best. A significant strength is that this study overcame several barriers to reach these students including school board gatekeeping, student attendance, mobility, and mistrust of systems. Those students feeling most connected to the CSS staff likely participated. We cannot know what knowledge would have been added by those who did not participate.

## Conclusion

This constructivist grounded theory study contributes to the literature by explicating the importance of connection for excluded students and the ways that unacknowledged exposure to adversity can block this connection. The range of experiences endorsed through expanded ACE inventories must be acknowledged and understood through a trauma-informed culturally attuned framework. Moreover, schools must be adequately resourced and safe for students and staff. This includes fostering knowledge, skills, time, and support to build connections with all students, families and communities, particularly those most likely to be impacted by expanded forms of adversity.
